# A New Improved Method for Assessing Brain Deformation after Decompressive Craniectomy

**DOI:** 10.1371/journal.pone.0110408

**Published:** 2014-10-10

**Authors:** Tim L. Fletcher, Angelos G. Kolias, Peter J. Hutchinson, Michael P. F. Sutcliffe

**Affiliations:** 1 Department of Engineering, University of Cambridge, Cambridge, United Kingdom; 2 Division of Neurosurgery, Department of Clinical Neurosciences, Addenbrooke’s Hospital and University of Cambridge, Cambridge, United Kingdom; University of Nebraska Medical Center, United States of America

## Abstract

**Background:**

Decompressive craniectomy (DC) is a surgical intervention used following traumatic brain injury to prevent or alleviate raised intracranial pressure. However the clinical effectiveness of the intervention remains in doubt. The location of the craniectomy (unilateral or bifrontal) might be expected to change the brain deformation associated with the operation and hence the clinical outcome. As existing methods for assessing brain deformation have several limitations, we sought to develop and validate a new improved method.

**Methods:**

Computed tomography (CT) scans were taken from 27 patients who underwent DC (17 bifrontal patients and 10 unilateral patients). Pre-operative and post-operative images were processed and registered to determine the change in brain position associated with the operation. The maximum deformation in the herniated brain, the change in volume and estimates of the craniectomy area were determined from the images. Statistical comparison was made using the Pearson’s correlation coefficient *r* and a Welch’s two-tailed *T*-test, with statistical significance reported at the 5% level.

**Results:**

There was a reasonable correlation between the volume increase and the maximum brain displacement (*r* = 0.64), a low correlation between the volume increase and the craniectomy area (*r* = 0.30) and no correlation between the maximum displacement and the craniectomy area (*r* = −0.01). The maximum deformation was significantly lower (*P*  = 0.023) in the bifrontal patients (mean = 22.5 mm) compared with the unilateral patients (mean = 29.8 mm). Herniation volume was significantly lower (*P* = 0.023) in bifrontal (mean = 50.0 ml) than unilateral patients (mean = 107.3 ml). Craniectomy area was not significantly different for the two craniectomy locations (*P* = 0.29).

**Conclusions:**

A method has been developed to quantify changes in brain deformation due to decompressive craniectomy from CT images and allow comparison between different craniectomy locations. Measured displacement is a reasonable way to characterise volume changes.

## Background

Traumatic brain injury (TBI) can cause swelling in the brain leading to uncontrolled raised intracranial pressure (ICP). This in turn can lead to death or severe brain damage. Therefore, reducing raised ICP is an important factor in treatment of TBI. There are two general methods used to counter this pressure, medical or surgical therapies [1]. If medical management is unsuccessful in lowering ICP then a surgical procedure, decompressive craniectomy (DC), may be undertaken. This is usually referred to as secondary DC in order to differentiate it from primary DC which is undertaken when evacuating an intracranial haematoma in the acute phase [2]. In this operation a section of skull is removed allowing the brain to expand outside the skull and so relieve the pressure. There has been renewed interest in DC over recent years [3], but the effectiveness of the treatment remains in doubt [3]–[6].

There are two standard forms of DC, the bifrontal and unilateral craniectomy, details are reviewed in [7]. These differ in terms of the location of the region of skull which is removed. Currently there is no consensus on the optimal location of the craniectomy, although unilateral craniectomy is the more common [8]. Surgical decisions on location of the DC are taken based on the presence of clinical features in the brain with no consideration of the geometric differences which are inherent between the two options. These decisions will be taken depending on factors such as midline shift (shifting of the brain towards one side), and any swelling present in pre-op CT scans [2].

It is currently a matter of debate whether the location of the craniectomy (either unilateral or bifrontal) might change the brain deformation associated with the operation and hence affect the clinical outcome. Measurement of the deformation of the brain post-craniectomy often follows the method proposed by Flint et al [9]. This method allows direct extraction of results from CT scans with no further processing required but suffers from significant deficiencies as discussed below. Specifically the Flint method is not appropriate for comparing deformation after bifrontal and unilateral craniectomies due to the differences in the geometry of the brain in the two locations. The aim of this paper is to improve on the Flint method to determine brain deformation as applied to DC and apply the method developed to a set of patient data to determine the effect of craniectomy location on brain displacement. In subsequent work we aim to examine the hypothesis that these deformation measures, along with clinical factors, can contribute to an improved prediction of clinical outcome and hence lead to a better understanding of how to optimise treatment and improve clinical outcome.

## Methods

### Patients

Data from 27 patients were used in the study, taking CT scans acquired during routine clinical care. Anonymised clinical data were collected in the course of the RESCUEicp study (Randomised Evaluation of Surgery with Craniectomy for Uncontrollable Elevation of intracranial pressure trial - ISRCTN66202560) [3] and from clinical audit of patient care in the Neurosciences Critical Care Unit/Neurosurgical Unit of Addenbrooke’s Hospital. Ethical approval for the RESCUEicp study has been obtained from the UK Multi Centre Research Ethics Committee (Eastern Region) and the clinical audit has been registered and approved by the Clinical Audit Department, Addenbrooke’s Hospital. No consent was obtained for the specific analysis described in this paper. All records/information were anonymised and de-identified prior to analysis.

The demography of the patients is summarised in Table S1, with their condition categorised using the Modified Marshall CT grade [10] detailed in Table S2. All patients had a severe traumatic brain injury with an abnormal CT image of the head and underwent a secondary DC operation (17 bifrontal and 10 unilateral craniectomies). Patients were ventilated and managed in the NCCU with a tiered therapeutic protocol aiming for an ICP<25 mmHg and cerebral perfusion pressure (CPP) around 60–70 mmHg.

Patients had a pre-op and post-op CT scan; the interval between these scans is given in Table S1. There is no statistical difference in the interval between pre-op and post-op CT scans for the two craniectomy locations.

The mean age of patients for bifrontal and unilateral craniectomies is 26 (±8.5 standard deviation) and 39.5 (±16.7 standard deviation), respectively. The difference in means is statistically significant (*P* = 0.037). While it has been shown that the volume of the brain decreases with age [11], this decrease is more marked over the age of 70 which is well above the mean ages of either the bifrontal or unilateral craniectomy populations. Therefore, it is unlikely that this difference in age will affect the brain deformation results.

### Flint method to determine deformation

**Figure 1:**
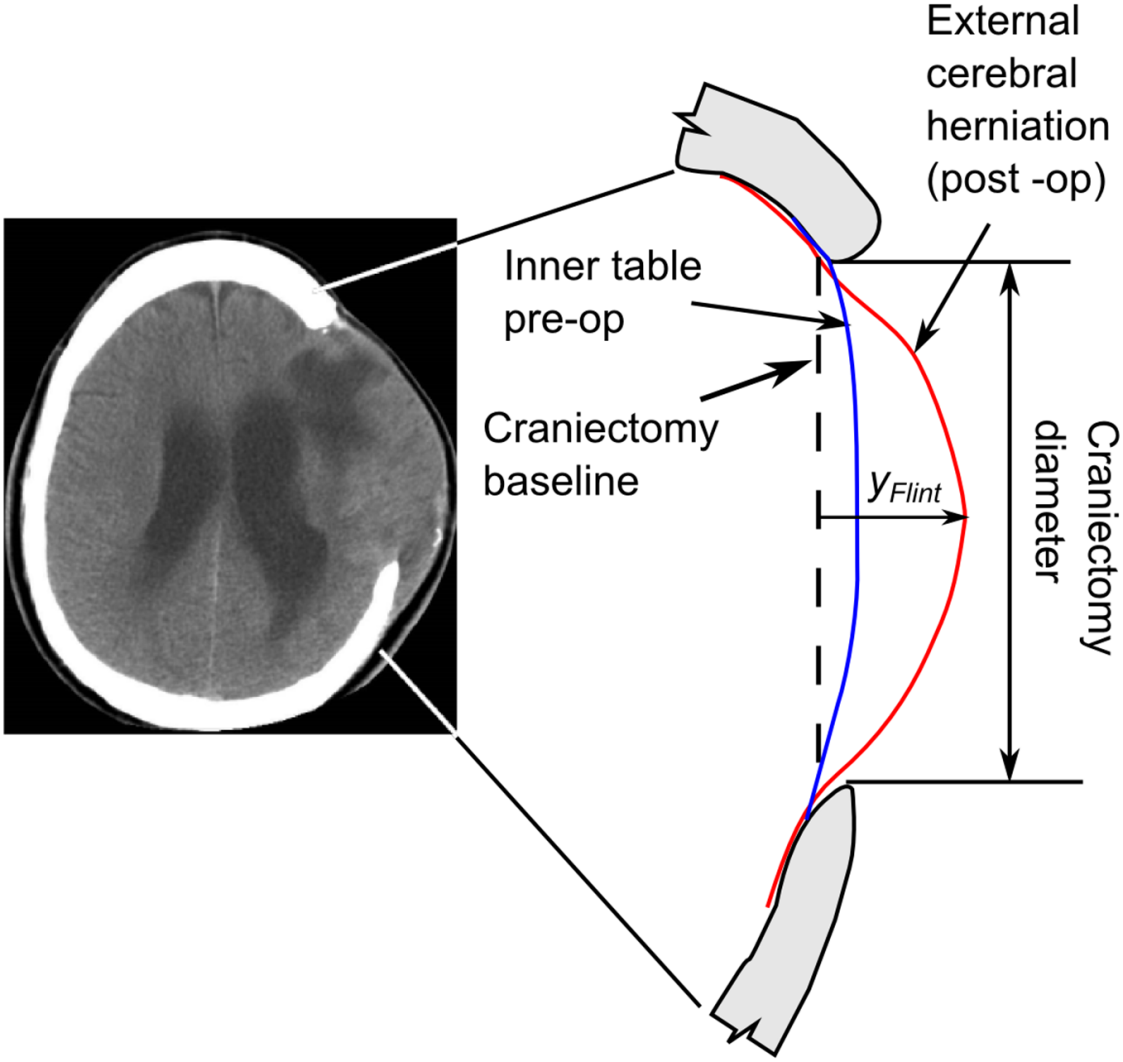
Geometry changes in the brain due to decompressive craniectomy.

The method typically used to measure displacement values [12] is that described in Flint et al. [9], see Figure 1. The size of the external cerebral herniation (ECH) is taken as the distance from the baseline of the craniectomy to the surface of the brain using a post-op CT scan, identified as *y_Flint_* in Figure 1. The baseline of the craniectomy is defined as the edge of the craniectomy opening in a single CT slice and is chosen for the slice with the maximum craniectomy diameter. For lateral craniectomy this method provides a simple measure to compare the ECH between patients. However it overestimates the actual displacement since it does not take account the pre-op shape of the brain. Moreover accurate comparison between bifrontal and lateral craniectomies is not possible with this method as a small change in the width of the craniectomy in a bifrontal craniectomy can cause a large change in the location of the base line due to the shape of the skull, leading to large changes in ECH values.

Whilst the Flint method has the advantage of being simple and requiring only post-operative data, it does not provide a direct measure of the brain deformation or a reliable method of comparing different DC locations. Hence the Flint method was modified as described below to create a measure of the deformation which can be used for a comparison between bifrontal and unilateral craniectomy openings and which can be automated. The key aspect which allows this comparison is determination of the deformation of the surface of the brain with respect to its pre-op location.

### Image processing to identify deformed shape

Because a patient who needs a DC has high ICP, the brain tends to be pushed to the inner surface of the skull, the inner table. Therefore, the pre-operative location of the surface of the brain is defined as the inner table of the skull. This definition of the surface of the brain makes identification of this surface straightforward in the pre-operative scan volume.

Pre-operative scans were rigidly registered to the post-operative scans in 3D Slicer [13]–[15] using the skull as a fixed reference, having first applied a threshold to isolate the skull. The re-orientated pre-op volume was re-sampled to match the post-operative orientation and slice locations such that corresponding slices existed in the pre-op and post-op volumes. Interpolation on to the new orientation follows a *β-*spline approach in 3D Slicer. The *β*-spline approach is widely used for image interpolation after transformations and the methods used here are based on those in [16]. In general, the post-op volumes were obtained with little or no gantry tilt, which affects the orientation of the slices (see Appendix S1 for a discussion of this factor). This means that the pre-op volume, once registered, will be in an axial orientation (disregarding any issues with patient head orientation).

After the registration step, the pre-op scan was output from 3D Slicer in the same orientation as the post-op scan. Equivalent slices in the pre-op and post-op scan are therefore produced.

The inner table (pre-op and post-op) and the edge of the deformed brain (post-op) were located by first adjusting the window and level parameters to accentuate the brain edge. This was followed by statistical region merging in Fiji [17], [18] and a thresholding step to eliminate those regions which were not required. Then an active-contour method in Fiji [17], [19] was used to create a clean binary mask with no “holes” in the brain region. This step helps simplify the volume calculations. The edge of the mask, located using a standard edge detection algorithm, was output as a series of co-ordinates for analysis and measurement of the deformation.

### Extraction of deformation

**Figure 2:**
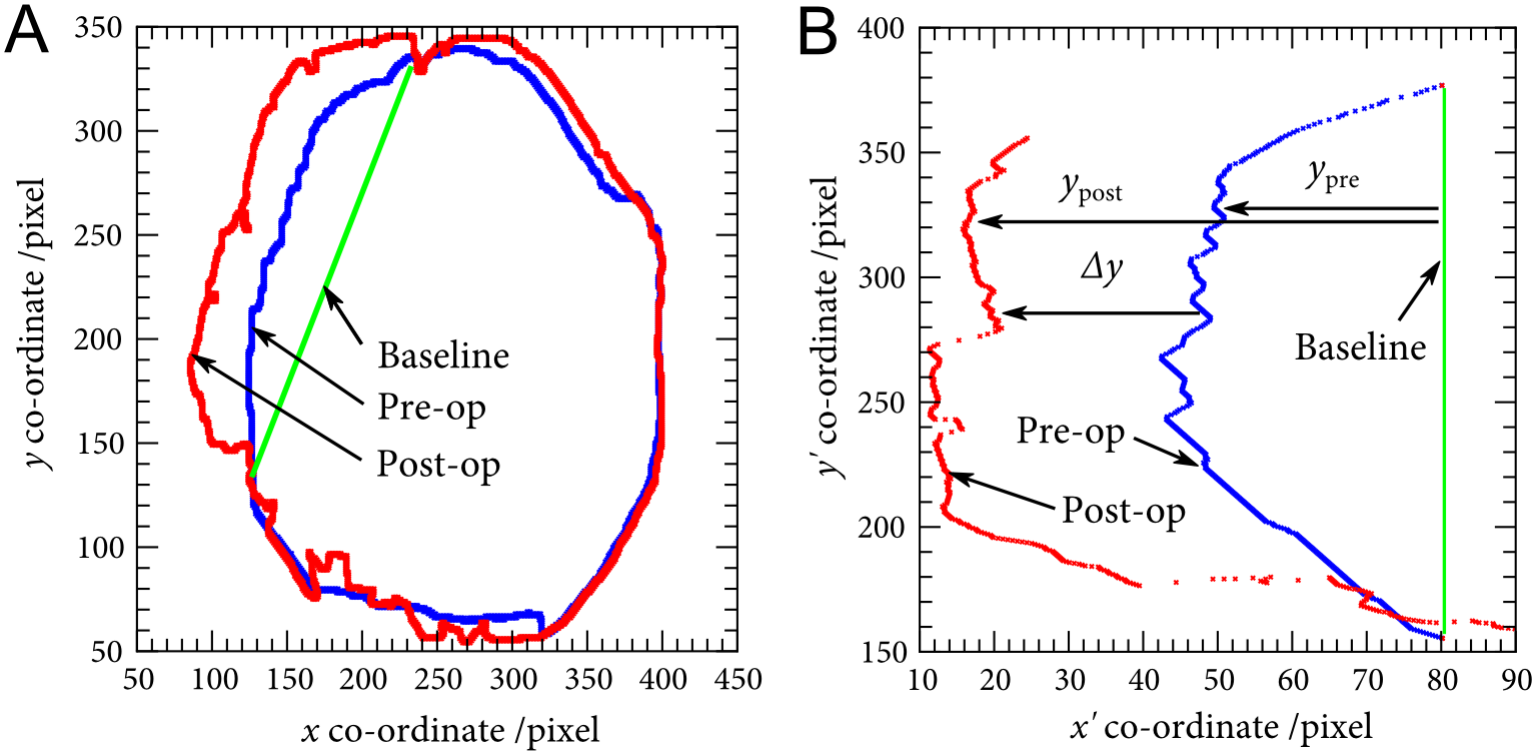
Identification of deformation from pre-op and post-op CT scans in a sagittal plane. A Outline of the brain edge, B Deformation at the external cerebral herniation. Note that the *x* scale is exaggerated in B.



To determine the brain deformation associated with DC, corresponding pre-op and post-op images were selected from the re-sampled pre-op scan and the original post-op scan. Figure 2A illustrates a typical pair of such scans along with the craniectomy baseline determined from the pre-op scan. The edges of the brain identified from segmentation were analysed to calculate the deformation at an orientation normal to the craniectomy baseline, as shown in Figure 2B (note that the scale in the direction normal to the baseline has been exaggerated in this figure). The difference between the pre-op and post-op margins gives the brain displacement *Δy*, defined as:

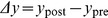
(1)By including the pre-op shape of the brain, this approach does not over-estimate craniectomy deformation as per the Flint method [9], which by contrast take *y*_Flint_ = *y*_post_ and so fails to take into account the pre-op shape of the brain.

The above measure of deformation was calculated for all points along the ECH at all *z*-locations in the CT scan volume. It provides a more complete measure of the deformation of the brain post-craniectomy, particularly the region in the ECH. The maximum displacement *Δy_max_* in the ECH was identified for each patient to allow inter-patient comparisons to be made.

The volume of the ECH was measured in the following manner. The masked volumes of the pre and post op brain were imported into 3D Slicer as a label map. This label map can be analysed using the label statistics module [20]. The label statistics module outputs the volume of the label map in question and the difference between the pre-op and post-op volume is considered to be the herniated volume. The volume analysis is similar in method to that in [21]. In a few cases issues with the CT images prevented either volume or deformation being obtained, as indicated in Table S1.

We view both the herniated volume and displacement as outputs of the surgical procedure which, along with the clinical outcome, are functions of clinical features including ICP and the details of the location and size of the craniectomy. It is anticipated that either or both of these measures of deformation might correlate with clinical outcome and hence be useful indicators of deformation changes.

### Estimate of craniectomy area

The shape of the craniectomy openings is complex and differs between craniectomy locations. The uni-lateral may be approximated as an ellipse in most cases, but the bifrontal craniectomy is a complex three dimensional opening. Two ways were used to determine the craniectomy area. The first method used 3D Slicer to create a 3D model from the CT images. Bone was identified using an intensity threshold, and the difference between pre-op and post-op models was used to calculate the change in skull surface area and hence the surface area of the craniectomy opening. However this method was found to be unreliable, reducing the number of useful patient data significantly. Hence an alternative simple estimate of the craniectomy area was made taking the product of the maximum height and width of the opening, as determined from the scans. This estimate of the area, assuming a rectangular shape, is a constant factor of 4/*π* = 1.27 greater than an estimate based on an elliptical shape. In most cases the actual shape is between these two extremes, and so the differences associated with this approximation are acceptable.

### Statistical analysis

Statistical comparison was made using the Pearson’s correlation coefficient *r* and unpaired, unequal-variance Welch’s two-tailed *T*-tests, with statistical significance reported at the 5% level. The probability *P* for the correlation coefficient tests the null hypothesis that the regression coefficient equals zero.

## Results and Discussion

**Figure 3:**
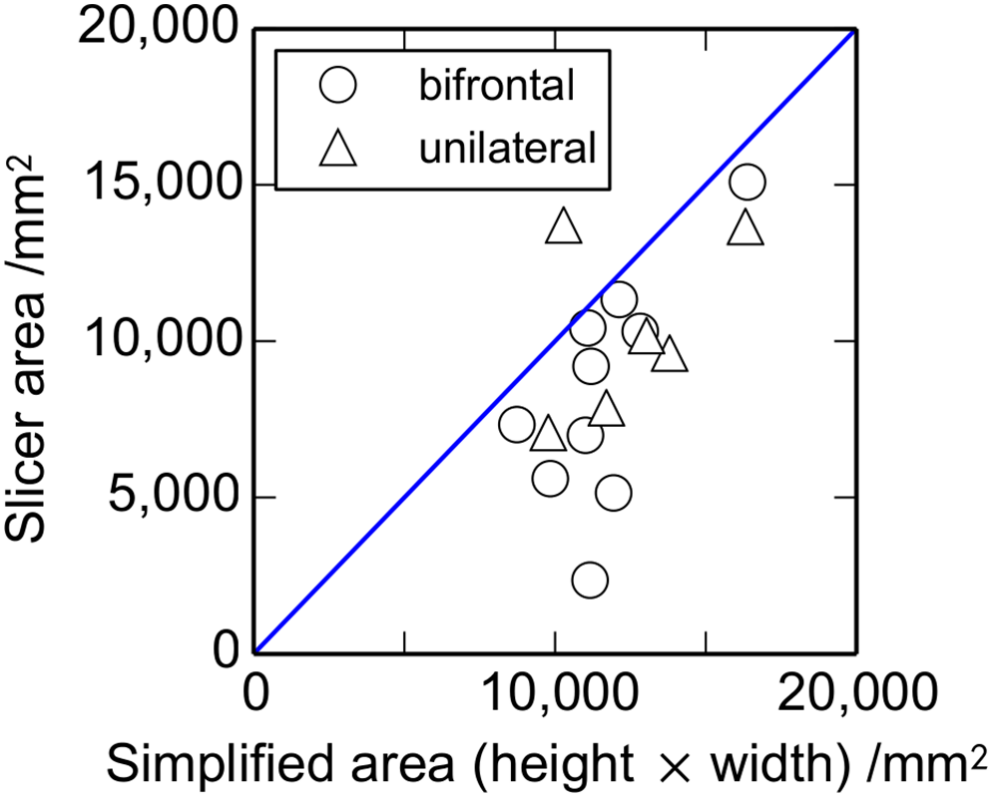
Comparison of craniectomy area estimates. Results for the simplified estimate of the craniectomy area using the height × width are compared with an estimate from a model using 3D Slicer (*n* (bifrontal) = 10, *n* (unilateral) = 6). The diagonal line corresponds to the two area estimates being equal.

Figure 3 compares estimates of the craniectomy area using the simple estimate as the height times the width of the opening with the estimate derived from the 3D Slicer model of the skull pre- and post-op. All but one of the simplified area results are larger than the corresponding Slicer area estimates. This systematic difference reflects the difference between the curved craniectomy opening and the rectangular area of the simplified estimate. The mean ratio of the simplified area to the Slicer area estimates equals 1.32, close to the value of 1.27 expected comparing a rectangular and elliptical shape. There is a reasonable correlation between the area estimates (*r* = 0.60, *P* = 0.013) confirming that the simplified area is a reasonable measure of craniectomy area. While the greater curvature of the bifrontal opening, as compared with the unilateral craniectomy, might be expected to give a larger surface area for a given height × width opening, the data of Figure 3 does not demonstrate this difference. From a biomechanics perspective, the opening area represented by the height × width estimate provides a useful measure of the space available for the brain tissue to herniate through. Given the uncertainty in some of the Slicer area estimates and the corresponding reduction in the number of valid patient data points, subsequent area measures use the height × width estimate. Correlations given below between the craniectomy area and the measures of brain deformation are not improved by using the Slicer area estimates.

**Figure 4:**
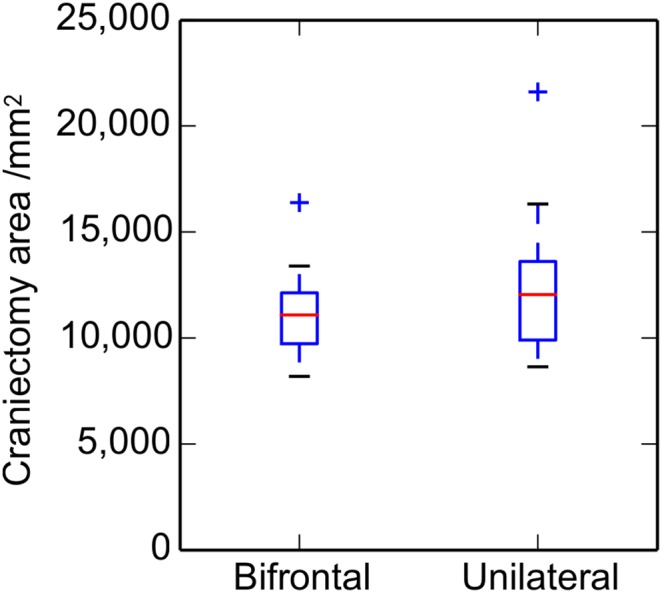
Variation with craniectomy location of the approximate craniectomy area. Box plot illustrating the results of the simplified area analysis (i.e. height × width) for bifrontal and unilateral craniectomies (*n* (bifrontal)  = 17, *n* (unilateral) = 10). The boxes show the median, upper and lower quartiles. Whiskers show the largest and smallest data points within 1.5 times the inter-quartile range of the upper and lower quartiles, respectively, and crosses identify outliers.

Box plots of the craniectomy areas estimated from the height times the width are shown in Figure 4, comparing results for the two craniectomy locations. The difference in mean areas is relatively small (11,200 and 12,700 mm^2^ for the bifrontal and unilateral cases, respectively) and there is no significant difference between the two sets of patients (*P* = 0.29). There is similarly no significant difference between the areas estimated using the Slicer models from the two sets of patient data (*P* = 0.25).

**Figure 5:**
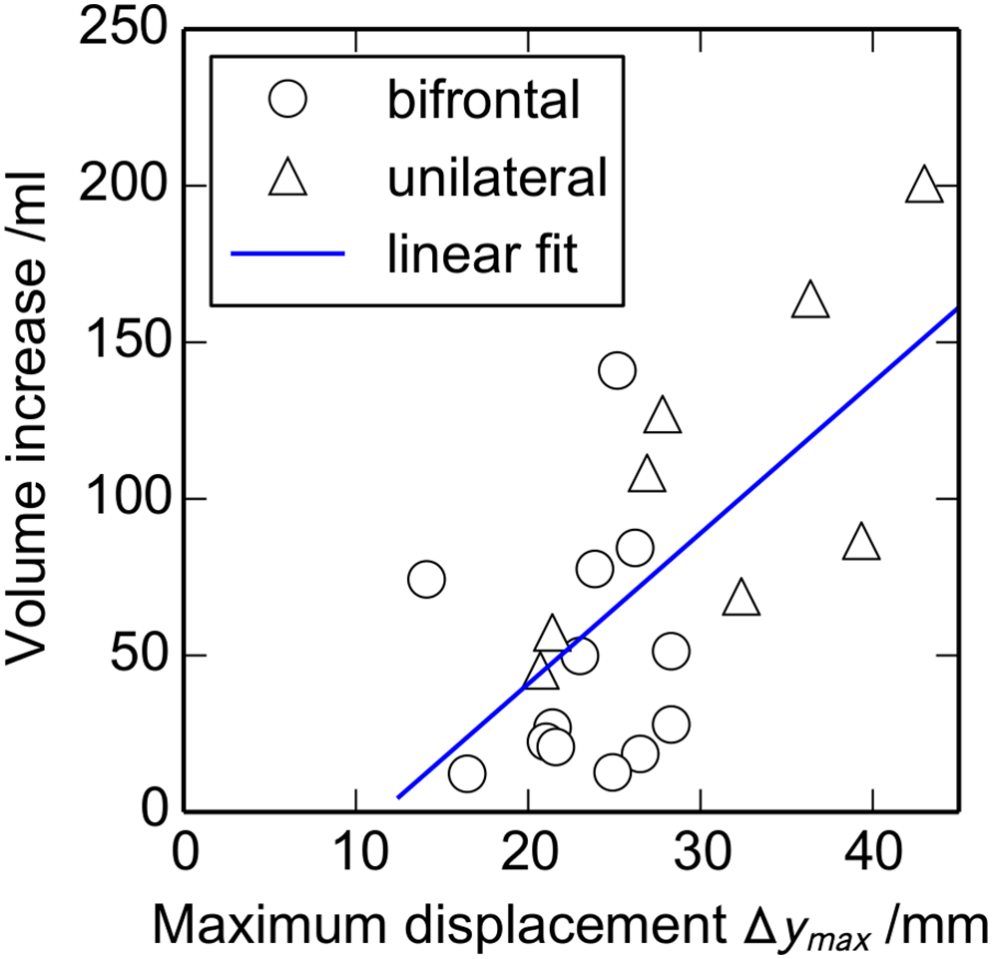
Relationship between the maximum displacement *Δy_max_* and the volume increase *ΔV*. The diagonal line is a least-squares linear fit, with a correlation *r = *0.64 (*n* (bifrontal) = 13, *n* (unilateral) = 8).

Figure 5 shows the relationship between maximum displacement and herniated volume. There is a reasonable correlation of *r* = 0.64 (*P* = 0.0016), indicating that bulge can be used as a reasonable predictor of the change in volume.

**Figure 6:**
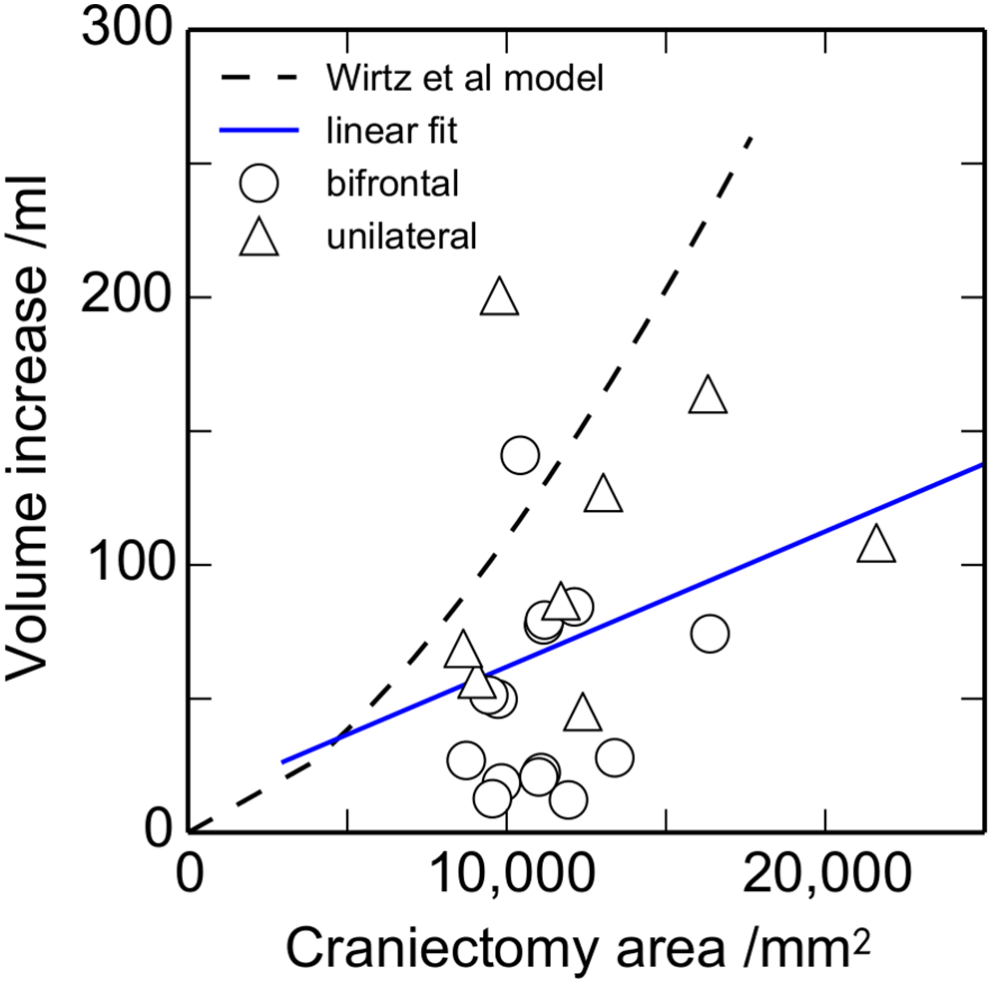
Relationship between the craniectomy area and the volume increase *ΔV*. The dashed line shows the prediction from the model of Wirtz et al. [22] and the solid line is a least-squares linear fit, with a correlation *r = *0.30 (*n* (bifrontal) = 14, *n* (unilateral) = 8).

Figure 6 shows the relationship between the craniectomy area and herniation volume. There is a low correlation between area and volume increase of *r* = 0.30 (*P* = 0.17). The volume increases with area, but not at the same rate as per the predictions of [22] included in the figure. This over-estimation of the model is probably due to the assumption in the model that the expansion is cylindrical. Although the model in [22] accounts for a restriction in the expansion near the craniectomy edge, this restriction would need to be increased in size to adequately match the relationship in practice.

To further assess the importance of the craniectomy area on herniated volume, a multiple regression has been undertaken, quantifying the correlation between the herniation volume and the displacement and surface area. As noted above, the correlation *r* between volume and displacement equals 0.64. Including additionally the surface area increases the correlation *r* between the volume and the displacement and area to 0.72. This rather modest improvement in correlation confirms that the effect of craniectomy area on the herniated volume is rather small.

**Figure 7:**
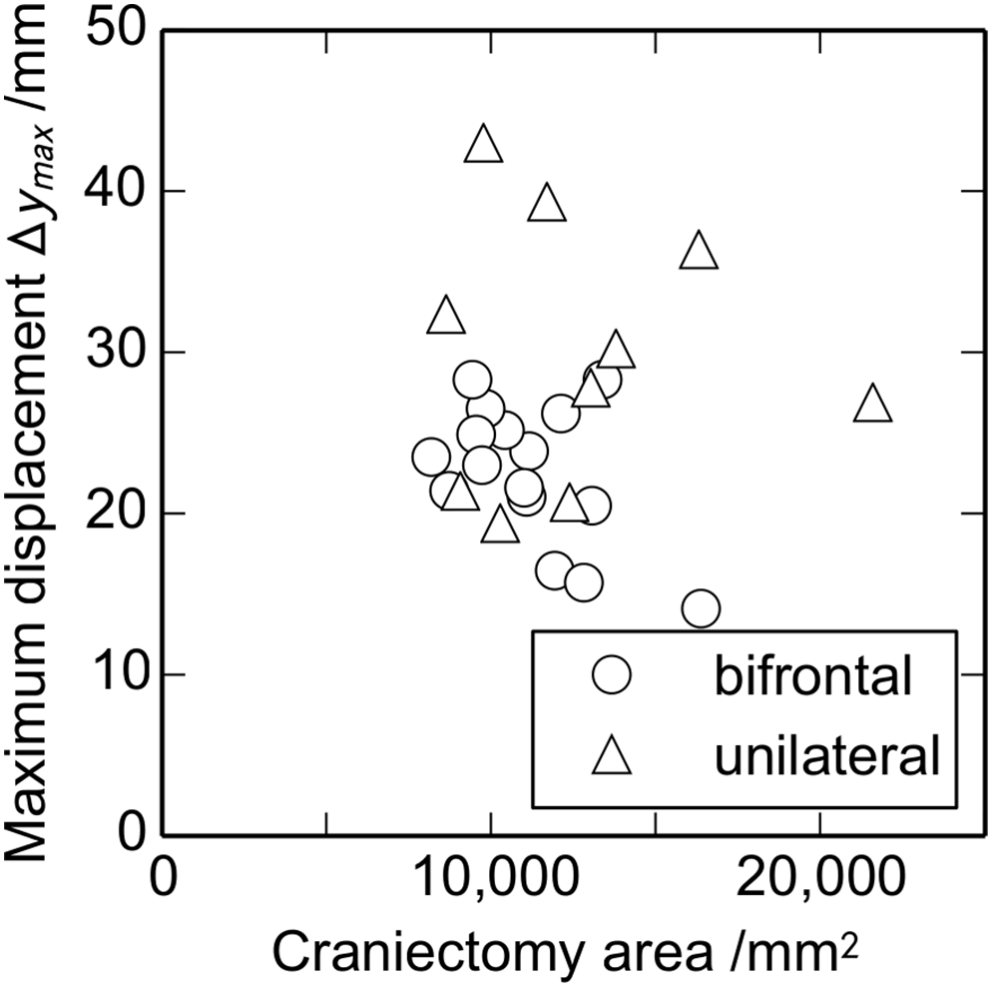
Relationship between the craniectomy area and the maximum displacement *Δy_max_* (*n* (bifrontal) = 16, *n* (unilateral) = 10).

Figure 7 shows the relationship between craniectomy area and maximum displacement. There is no correlation between these parameters (*r* = −0.011, *P* = 0.96). This lack of correlation contrasts with the equivalent result for volume versus area, Figure 6, which showed a low correlation of *r* = 0.30.

For a given ICP or degree of brain swelling we might expect the maximum displacement to depend on the craniectomy area selected by the surgeon. For example a larger area would hypothetically give a smaller deformation for a constant volume and shape of herniation. Or a larger area might give a larger deformation for a herniation represented by a model of the brain expanding under a constant internal pressure. But in fact results do not show a strong dependence on craniectomy area. This suggests that the expansion may be somewhere between the constant pressure and constant volume models, with the deformation details affected presumably by physiological and clinical factors post-op.

**Figure 8:**
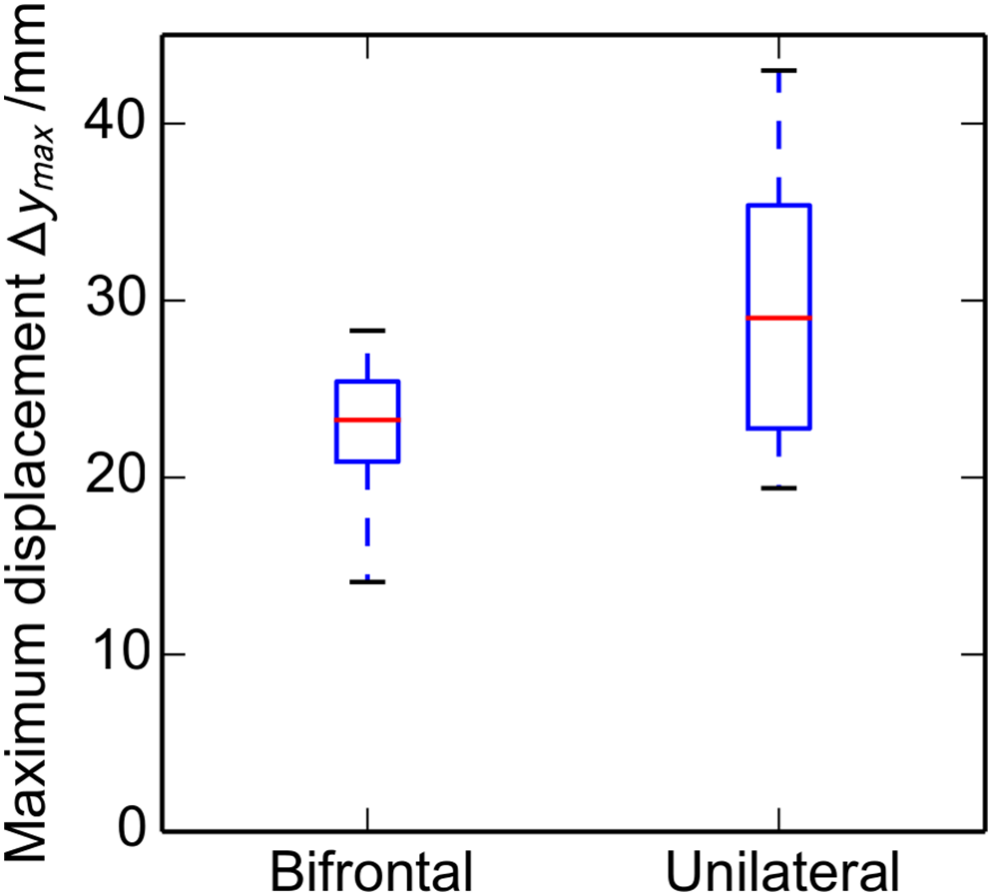
Variation with craniectomy location of the maximum displacement *Δy_max_* in the ECH. Box plot illustrating the results of the displacement analysis for bifrontal and unilateral craniectomies (*n* (bifrontal) = 16, *n* (unilateral) = 10). The median, upper and lower quartiles, and the range are shown.

Figure 8 shows boxplots comparing the maximum herniated displacement between the bifrontal and unilateral craniectomy patients. The mean displacements are significantly smaller for the bifrontal than the unilateral cases (*P* = 0.023), being 22.5 and 29.8 mm, respectively.

**Figure 9:**
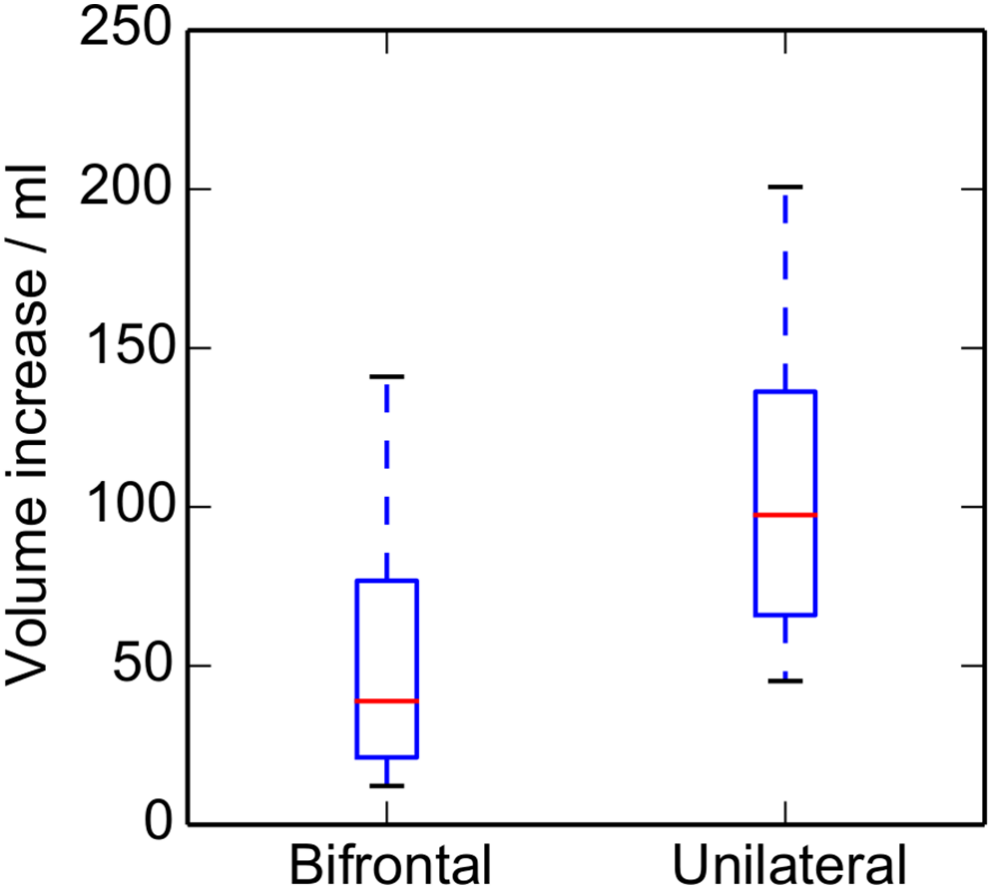
Variation with craniectomy location of the increase in herniation volume *ΔV* in the ECH. Box plot illustrating the results of the volume analysis for bifrontal and unilateral craniectomies (*n* (bifrontal) = 14, *n* (unilateral) = 8). The median, upper and lower quartiles, and the range are shown.

The corresponding results for the herniation volume increase are plotted in Figure 9. Again the mean volume increases are significantly smaller for the bifrontal than the unilateral cases (*P* = 0.023), being 50.0 and 107.3 ml, respectively.

The mean herniation volume for the unilateral cases in the current study of 107.3 ml (standard deviation: 54.2 ml) is within the range of values of 27–127 ml reported for the 6 lateral craniectomy patients in the study of von Holst et al. [21]. Since only lateral craniectomies are reported in [21], there is no corresponding comparison possible for the bifrontal cases.

These results for the volume change can be related to the maximum displacement *Δy_max_* for the lateral craniectomy assuming a simple model of the displacement. Assuming that the deformation varies parabolically with distance from the craniectomy edge through a circular craniectomy opening of radius *r*, the volume change *ΔV* is given by:
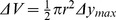
(2)Taking representative values for *r* of 35 mm and *Δy_max_* of 25 mm, the corresponding volume *ΔV* equals 48,000 mm^3^ or 48 ml. This compares reasonably well with the average measured volume for bifrontal and lateral craniectomy openings of 50.0 and 107.3 ml, respectively, giving further confidence in the estimated herniation volumes. Nevertheless it is suggested that it would be better to use the clinical measures of deformation and volume directly in a clinical comparison rather than geometric models such as equation 2 or Wirtz et al [22], which contain assumptions about the deformation not generally well supported by the data presented.

A method recently described in [21] uses the diffeomorphic demons method of non-rigid registration to ascertain displacement measures for each voxel in a 3-dimensional CT scan volume. In principle the method allows inter-patient comparisons to be drawn, both for lateral and bifrontal craniectomy openings. The method of [21] was adopted and applied to the scans used in this study. However poor results were obtained. This was attributed to errors associated with the large slice thicknesses of the CT scans, required to minimise the scan time which is a critical clinical factor for these patients. Despite this negative result, the method described in [21] should be considered for future analysis of DC, particularly with the advent of faster CT scans which could allow much smaller slice thickness scans to be collected even in DC patients.

## Conclusions

A method has been developed to quantify changes in brain deformation due to decompressive craniectomy from CT images. It is suggested that both maximum displacement and herniated volume could be used to correlate deformation with clinical outcome.

The correlation between maximum displacement of the brain and the change in volume was reasonable, confirming that the simpler displacement method may be a reasonable clinical marker for deformation instead of volume.

There was a low correlation of herniated volume with craniectomy area, and no correlation of maximum displacement with area. These results and the relatively poor agreement of results with the model of [22] suggest that it would be preferable to measure herniated volume directly rather than rely on an estimate based on a simple geometrical model.

Both the maximum displacement and the change in volume were significantly smaller for bifrontal than unilateral cases.

## Supporting Information

Table S1 Patient demography: * - no volume data, + - no displacement data.
(PDF)

Table S2 **Modified Marshall CT grading system** [10] **.**
(PDF)

Appendix S1 **Gantry tilt correction.**
(PDF)
