# Supplementary material for: A New Improved Method for Assessing Brain Deformation after Decompressive Craniectomy
Source: PLoS One. 2014 Oct 10;9(10):e110408. doi: 10.1371/journal.pone.0110408 (PMC4193893; doi:10.1371/journal.pone.0110408)
Supplement: Table S1 — Patient demography: * - no volume data, + - no displacement data. (PDF) [file pone.0110408.s001.pdf]

A new improved method for assessing brain deformation after decompressive craniectomy

Tim L Fletcher, Angelos G Kolias, Peter J Hutchinson, Michael PF Sutcliffe

Table S1. Patient demography: * - no volume data, + - no displacement data.

Gender	Age (years)	Pre-DC Marshall grade	Location	Interval between scans (days)
M	17	3	Bifrontal	1
M *	39	2d	Bifrontal	1
F	18	3	Bifrontal	1
M	31	3	Bifrontal	5
F	17	3	Bifrontal	2
M *	19	4	Bifrontal	11
M	20	3	Bifrontal	7
M	18	6c	Bifrontal	1
F +	21	2c	Bifrontal	5
M	27	2c	Bifrontal	1
M	27	2d	Bifrontal	1
F	25	3	Bifrontal	1
M *	25	2c	Bifrontal	3
M	36	3	Bifrontal	2
M	29	3	Bifrontal	5
M	29	2d	Bifrontal	2
M	47	6d	Bifrontal	10
M	44	6c	Unilateral	1
M	50	2d	Unilateral	4
F	12	2c	Unilateral	1
F	17	5a	Unilateral	1
M	45	4	Unilateral	1
M *	26	3	Unilateral	1
M	54	6c	Unilateral	3
F	32	3	Unilateral	3
F *	59	5b	Unilateral	3
F	56	3	Unilateral	6
