# Supplementary material for: A New Improved Method for Assessing Brain Deformation after Decompressive Craniectomy
Source: PLoS One. 2014 Oct 10;9(10):e110408. doi: 10.1371/journal.pone.0110408 (PMC4193893; doi:10.1371/journal.pone.0110408)
Supplement: Table S2 — Modified Marshall CT grading system [10] . (PDF) [file pone.0110408.s002.pdf]

# A new improved method for assessing brain deformation after decompressive craniectomy

Tim L Fletcher, Angelos G Kolias, Peter J Hutchinson, Michael PF Sutcliffe

**Table S2.** Modified Marshall CT grading system [10]

| | | | |
|---|---|---|---|
| **I** | Diffuse Injury | No visible pathology | 1 |
| **II** | Diffuse Injury (with present cisterns, midline shift 0-5 mm and/or small (<25 cc) high or mixed density lesions | No lesions | 2a |
| | | Only one lesion | 2b |
| | | ≥ 2 unilateral lesions | 2c |
| | | Bilateral lesions | 2d |
| **III** | Diffuse injury and swelling | **I–II** + compressed or absent cisterns | 3 |
| **IV** | Diffuse injury and shift | **I–III** + midline shift > 5 mm | 4 |
| **V** | Evacuated mass lesion | Extradural | 5a |
| | | Subdural | 5b |
| | | Intracerebral | 5c |
| | | ≥ 2 intra + extracerebral | 5d |
| **VI** | Non-evacuated mass lesion (>25 cc) | Extradural | 6a |
| | | Subdural | 6b |
| | | Intracerebral | 6c |
| | | ≥ 2 intra + extracerebral | 6d |

## Reference

10. Marshall LF, Marshall SB, Klauber MR, Van Berkum Clark M, Eisenberg H et al. (1992) The diagnosis of head injury requires a classification based on computed axial tomography. Journal of Neurotrauma 9: S287–92.
