# Supplementary material for: A New Improved Method for Assessing Brain Deformation after Decompressive Craniectomy
Source: PLoS One. 2014 Oct 10;9(10):e110408. doi: 10.1371/journal.pone.0110408 (PMC4193893; doi:10.1371/journal.pone.0110408)
Supplement: Appendix S1 — Gantry tilt correction. (PDF) [file pone.0110408.s003.pdf]

A new improved method for assessing brain deformation after decompressive craniectomy

Tim L Fletcher, Angelos G Kolias, Peter J Hutchinson, Michael PF Sutcliffe

Appendix S1. Gantry tilt correction.

Gantry tilt describes the orientation angle of the CT scan detector gantry. This is sometimes non-zero in order to minimise radiation to vital organs, as illustrated in Figure 1 A and B. A scan with a non-zero gantry tilt angle will be sheared as illustrated in Figure 1C. Gantry tilt angle was corrected by applying a shear transformation in the same manner as [1, 2]. Each slice of the sheared volume is transformed by an affine transformation matrix *T*, which is of the form:

$$T = \begin{bmatrix} 1 & 0 & 0 \\ 0 & 1 & 0 \\ 0 & s\tan(\alpha) & 1 \end{bmatrix} \quad (1)$$

where s is the slice thickness and α the gantry angle as defined in Fig. 1B. The output from the transformation is a sheared volume which is corrected for gantry tilt, but with "axial" slices on an inclined plane to the true axial orientation, see for examples the corrected image of Fig. 1D.

References

1. von Holst H, Li X, Kleiven S (2012) Increased strain levels and water content in brain tissue after decompressive craniotomy. Acta Neurochirurgica 154(9):1583-1593

2. Li X (2012) Finite element and neuroimaging techniques to improve decision making in clinical neuroscience. PhD Thesis, KTH Stockholm.

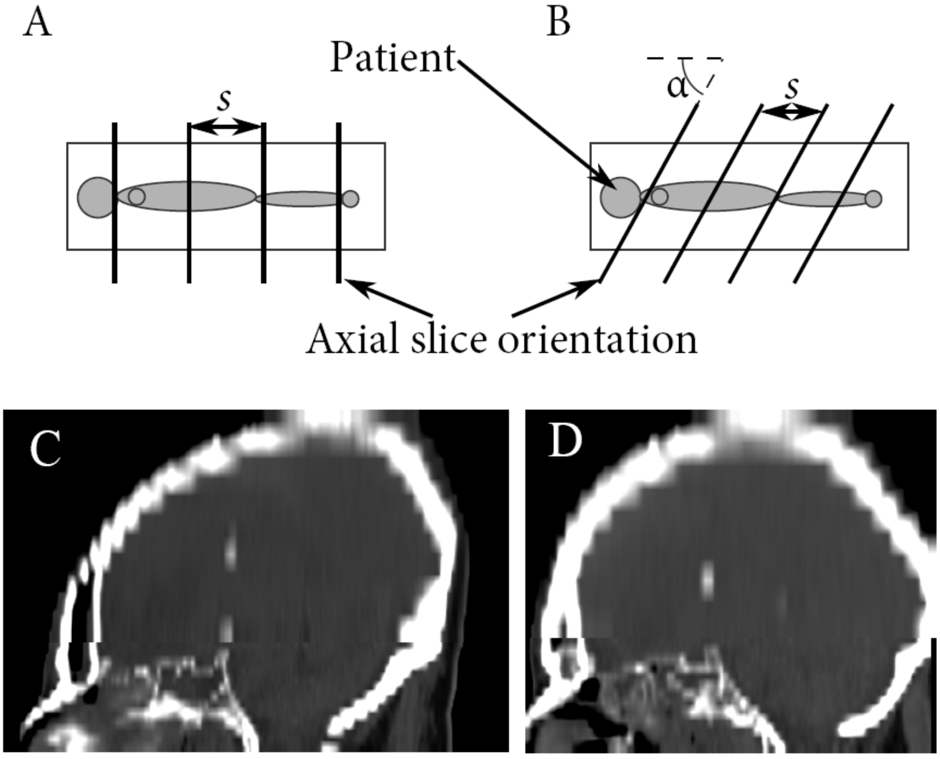

Figure 1. Illustration of the effect of gantry tilt. A Imaging with zero gantry angle; B imaging with a gantry tilt angle α; C a CT scan uncorrected for gantry tilt; D scan corrected for gantry tilt.
